# Localization and Aggregation of Honokiol in the Lipid Membrane

**DOI:** 10.3390/antiox13081025

**Published:** 2024-08-22

**Authors:** José Villalaín

**Affiliations:** Institute of Research, Development, and Innovation in Healthcare Biotechnology (IDiBE), Universidad “Miguel Hernández”, E-03202 Elche, Alicante, Spain; jvillalain@umh.es; Tel.: +34-6-4889-1404

**Keywords:** plasma membrane, molecular dynamics, aging, antioxidant

## Abstract

Honokiol, a biphenyl lignan extracted from bark extracts belonging to Magnolia plant species, is a pleiotropic compound which exhibits a widespread range of antioxidant, antibacterial, antidiabetic, anti-inflammatory, antiaggregant, analgesic, antitumor, antiviral and neuroprotective activities. Honokiol, being highly hydrophobic, is soluble in common organic solvents but insoluble in water. Therefore, its biological effects could depend on its bioactive mechanism. Although honokiol has many impressive bioactive properties, its effects are unknown at the level of the biological membrane. Understanding honokiol’s bioactive mechanism could unlock innovative perspectives for its therapeutic development or for therapeutic development of molecules similar to it. I have studied the behaviour of the honokiol molecule in the presence of a plasma-like membrane and established the detailed relation of honokiol with membrane components using all-atom molecular dynamics. The results obtained in this work sustain that honokiol has a tendency to insert inside the membrane; locates near and below the cholesterol oxygen atom, amid the hydrocarbon membrane palisade; increases slightly hydrocarbon fluidity; does not interact specifically with any membrane lipid; and, significantly, forms aggregates. Significantly, aggregation does not impede honokiol from going inside the membrane. Some of the biological characteristics of honokiol could be accredited to its aptitude to alter membrane biophysical properties, but the establishment of aggregate forms in solution might hamper its clinical use.

## 1. Introduction

**Figure 1:**
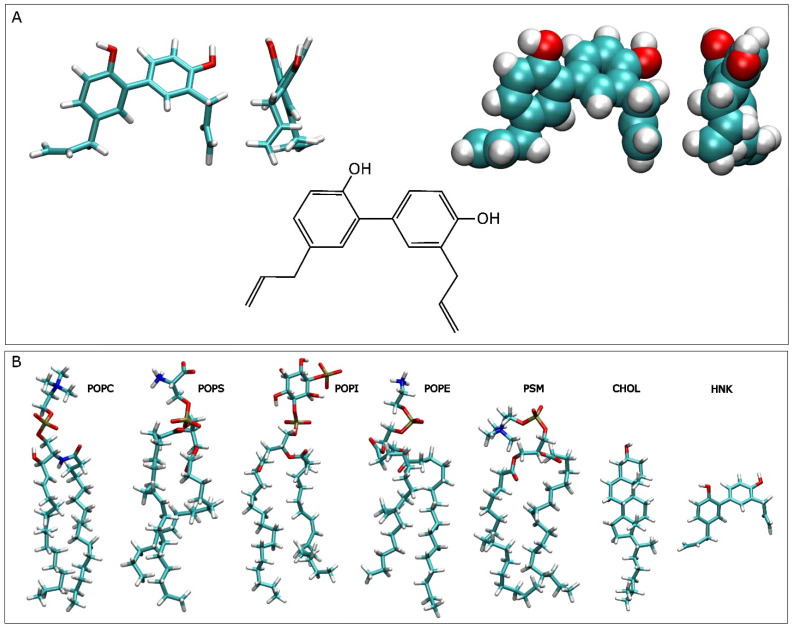
(**A**) Molecular and chemical structures of honokiol and (**B**) molecular structures of the lipid molecules used in this study: POPC, POPE, POPS, PI-3P, PSM, and CHOL. The molecular structure of honokiol is shown in (**B**) in order to compare its size with the membrane lipids.

Natural products are an important source of drug development for human and animal health [1,2,3]. Lignans are natural molecules, known as phytoestrogens, formed through the polymerization of two phenyl-propanoid units, prevailing in the dimer form [4,5,6]. Numerous lignans, having significant biological properties, have been revealed; one of the most characteristic ones is the lignan honokiol (HNK) (Figure 1A) [4,5,6]. HNK is a bisphenol neolignan abundant in the root bark of Magnolia plant species and distributed in many regions of the world [7,8]. HNK is a pleiotropic compound, is a commonly used ingredient in Asian homeopathic medicine, and is attributed to have an extensive range of benefits such as antibacterial, antidiabetic, anti-inflammatory, antioxidant, antiaggregant, analgesic, antitumor, antiviral and neuroprotective activities [7,9,10,11,12,13,14,15,16,17,18,19,20,21,22,23,24,25,26]. HNK has a highly hydrophobic character and is soluble in organic solvents but insoluble in water, having a relatively high phospholipid/water partition coefficient (XLogP3 value 5, data obtained from PubChem, https://pubchem.ncbi.nlm.nih.gov/compound/72303, accessed on 19 August 2024); the biological effects of HNK could depend on its bioactive mechanism. Its relatively high hydrophobicity has limited its clinical applications [9] but also permits it to pass the blood–brain barrier [7,27]. From the clinical point of view, HNK, a molecule with a low molecular weight (226.34 Da), is chemically characterized by one para-allyl-phenol and an ortho-allyl-phenol, which are interconnected by an ortho-para-C-C coupling. This coupling should allow interaction with a large variety of targets [28].

Organisms have developed antioxidant defence systems to respond to the existence of high contents of free radicals; antioxidants are the preferred mechanism for inhibition of the initiation and propagation of reactive oxygen species, [29,30,31]. Antioxidants should stop the loops between oxidative stress, protein misfolding, inflammation, and lipid peroxidation, which give rise to many health problems [31]. HNK has noteworthy antioxidant properties helping to counter oxidative stress by neutralizing free radicals [15,28,32,33,34]. In fact, it has been described that honokiol enhanced mitochondrial antioxidant capacity upon sirtuin activation through the AMPK/PGC-1α signalling pathway [35].

Although HNK has many bioactive properties and it has had a significant increase in attention in the last years, its effects at the level of the biological membrane are unknown, and therefore in-depth research on HNK is required to be able to evaluate its use in the clinic. Furthermore, since HNK is a hydrophobic molecule, it could insert inside the biological membrane and modulate membrane structure, altering biophysical properties. HNK’s interaction with the biological components of the membrane, both lipids and proteins, its location inside the membrane and its adjustments of membrane dynamics and structure, might be accountable for its effects on human health. Understanding the mechanism of action of HNK could open new perspectives for its therapeutic development or even help obtain similar molecules with improved effects. However, its clinical use could be partially blocked by its reduced solubility in water, i.e., the formation of aggregates. 

Full-atom molecular dynamics (MD) is suitable for knowing the molecular dynamics, structure, location, and interaction of molecules interacting with biological membranes [36,37,38]. I have used MD to determine the orientation and location of HNK in a plasma-like membrane and, simultaneously, to find out if there are any interactions with membrane components. The data can be generalised to the conditions that take place in the cell, i.e., to describe the mechanism of action of HNK at the membrane level. I have studied four dissimilar systems which contained different numbers of HNK molecules, both inside and outside the membrane (Table 1). The results sustain that HNK tends to insert into the membrane and tends to locate near and below the CHOL oxygen atom, amid the hydrocarbon chains of the phospholipids, increasing slightly their fluidity. Outstandingly, HNK tends to associate spontaneously in solution, forming aggregates, but aggregation does not impede HNK from inserting into the membrane. Some of the biological properties of HNK could be accredited to its aptitude to alter membrane biophysical properties, but the establishment of aggregate forms in solution might hamper its clinical use.

## 2. Materials and Methods

### 2.1. Molecular Dynamics Simulation

The MD parameters used in this study have been previously described [39,40,41,42]. MD simulations were carried out using NAMD version 3.0b2 with the CHARMM36 protein and lipid force fields. All simulations were carried out with a constant number of particles as an NPT ensemble at 1.0 atm and 310 K. The time step was 1 fs. Constant temperature was maintained by Langevin dynamics with a damping coefficient γ of 0.5 ps-1, and constant pressure was maintained by the Nosé–Hoover Langevin piston method. The standard PME method was used with periodic boundary conditions to calculate the long-range electrostatic interaction of the systems. To remove bad atomic contacts, all systems were minimized for 150,000 steps; afterwards, they were equilibrated for 10 ns. The trajectories were run for 1 µs (Table 1). A Bull X410 computer node with 2 Xeon Platinum v5 processors with 768 Gb of RAM and 4 Nvidia V100 GPUs was used to obtain the molecular dynamic runs.

### 2.2. Molecular Dynamics Technical Specifications

**Figure 2:**
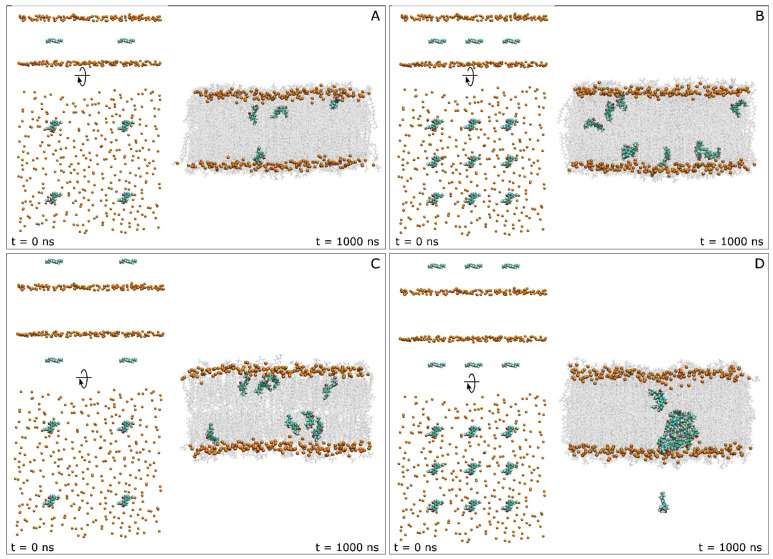
Lateral and apical views of the initial, t = 0 ns, and lateral view of the final, t = 1 µs, dispositions of (**A**) system 1, (**B**) system 2, (**C**) system 3, and (**D**) system 4. The location of the HNK molecules in each one of the systems is also displayed. The HNK molecules and the phosphorous atoms of the phospholipids, which define the upper and lower boundaries of the membrane, are drawn in VDW representation. Lipids at t = 1 µs are drawn in transparent licorice representation. The lipid and water molecules and the chloride and sodium ions have been removed for clarity. The formation of a big oligomer can be clearly observed in system 4 (**D**).

I have analysed 4 model membrane systems having a lipidic composition like that of the plasma membrane [43,44] (Table 1). Each one of the systems contained a different concentration of HNK molecules as well as different locations of HNK molecules, both outside and inside the membrane (Table 1 and Figure 1). The membrane systems were made using Charmm-Gui (http://www.charmm-gui.org accessed on 19 August 2024, [45]). The systems contained water in excess [46] (Table 1). Each one of the systems was enclosed in a rectangular box and contained HNK, a membrane bilayer, a concentration of 150 mM NaCl, i.e., physiological conditions, and water (Table 1) [47,48,49]. All systems were neutral. The membrane system contained 144 molecules of 1-palmitoyl-2-oleoyl-sn-glycero-3-phosphocholine (POPC), 86 molecules of 1-palmitoyl-2-oleoyl-sn-glycero-3-phosphoethanolamine (POPE), 32 molecules of 1-palmitoyl-2-oleoyl-sn-glycero-3-phosphoserine (POPS), 28 molecules of 1-palmitoyl-2-oleoyl-sn-glycero-3-phosphoinositol-3-phosphorous (PI-3P), 60 molecules of N-stearoyl-D-erythro-sphingosylphosphorylcholine (PSM) and 150 molecules of cholesterol (CHOL) (Table 1) [43,44]. The relative molar percentage of the lipids was 28.8% for POPC, 17.2% for POPE, 6.4% for POPS, 5.6% for PI-3P, 12% for PSM and 30% for CHOL (Table 1) [43,44]. The whole number of lipids was 500, 250 in each membrane layer. The lipid chemical structures are shown in Figure 1B. The membrane general fluidity was increased by using one oleoyl hydrocarbon chain in POPC, POPE, POPS and PI-3P, the other one being a palmitoyl hydrocarbon chain [39,40,41,42]. PSM, in addition to containing a sphingosyl hydrocarbon chain, contained a palmitoyl one. The bilayer surface, i.e., the x–y plane, was perpendicular to the z-axis of the membrane. Both cross-sectional area and height of the simulation box were allowed to vary independently of each other without any restraints. The initial dimensions of the complete systems are shown in Table 1. The three-dimensional molecular structure of HNK, obtained from PubChem (https://pubchem.ncbi.nlm.nih.gov/ accessed on 19 August 2024) was adjusted and minimized using Discovery Studio 4.0 (Accelrys Inc., San Diego, CA, USA). The CHARMM General Force Field compatible stream files of HNK were accomplished using Charmm-Gui (http://www.charmm-gui.org [45]). The original systems at 0 ns are shown in Figure 2. Systems 1 and 2 contained 4 and 9 molecules of HNK, respectively, positioned at the middle of the membrane, whereas systems 3 and 4 contained 8 and 18 molecules of HNK, respectively, located in the water solvent, half of them at each side of the membrane (Table 1 and Figure 2). These systems are suitable to study the interaction of small bioactive molecules with membrane lipids [38,47,48,49]. My own preliminary studies using umbrella sampling simulations with HNK-like molecules show that their degree of insertion and interaction depends not only on the structure of the molecule, but also on membrane lipid composition and the degree of force used (see [50,51]). Therefore, in this work I have let the molecules interact with the membranes on their own.

### 2.3. Molecular Dynamics Analysis

Analyses and visualisations were made using VMD software 1.9.3 and plugins [38,39,40,52,53,54]. The VMD “Membplugin” [38,42,53,54] and VMD “Density Profile Tool” plugins [55] were used to obtain the corresponding lipid data. The order parameter, *S_CD_*, is defined as SCD=12 3 cos2 θ−1, where *θ* is the angle between the *C*–*D* vector and the bilayer normal plane, and the brackets represent an ensemble average. Hydrogen bonds were defined previously [38,42].

## 3. Results and Discussion

Understanding the HNK mechanism of action could unlock new perspectives for its therapeutic development or for therapeutic development of molecules similar to it. In order to be able to evaluate both the qualitative and quantitative behaviour of HNK molecules in the plasma membrane, different systems and arrangements have been used, that is, different arrangements of HNK location at the beginning of the simulation as well as different numbers of HNK molecules in the systems. For this reason, I have used four different membrane/HNK systems, differing by the number of as well as the location of the HNK molecules (Table 1 and Figure 2). System 1 contained four molecules of HNK at the central part of the membrane (Figure 2A); system 2 contained nine molecules of HNK at the central part of the membrane (Figure 2B); system 3 contained eight molecules of HNK at the middle of each water layer, four at each side (Figure 2C); and system 4 contained eighteen molecules of HNK at the middle of each water layer, nine at each side (Figure 2D). I believe that the number of systems used is appropriate to be able to evaluate the behaviour of HNK in the plasma membrane.

Variations in membrane thickness and lipid areas were applied to estimate the time to membrane equilibration [42,56,57]. The membrane thickness remained constant after ~75 ns for all four systems. For the last 30 ns they were practically identical, i.e., the values varied between 45 and 46 Å (Supplementary Table S1 and Supplementary Figure S1). These values are analogous to those described previously for CHOL-containing systems [58]. Lipid areas were practically constant after ~60 ns for all systems (Supplementary Figure S2). The average area for all the lipids for the last 30 ns of MD are shown in Supplementary Table S1, the lipid areas being comparable to those reported previously [54,58,59]. Consequently, all systems were equilibrated very early and reached a steady state after ~75 ns of simulation. No remarkable fluctuations were observed for these diluted systems having 500 lipid molecules when compared to each other: system 1 contained four HNK molecules, i.e., a lipid/HNK ratio of 125; system 2 contained nine HNK molecules, i.e., a lipid/HNK ratio of 55.5; system 3 contained eight HNK molecules, i.e., a lipid/HNK ratio of 62.5; and system 4 contained eighteen HNK molecules, i.e., a lipid/HNK ratio of 27.8.

**Figure 3:**
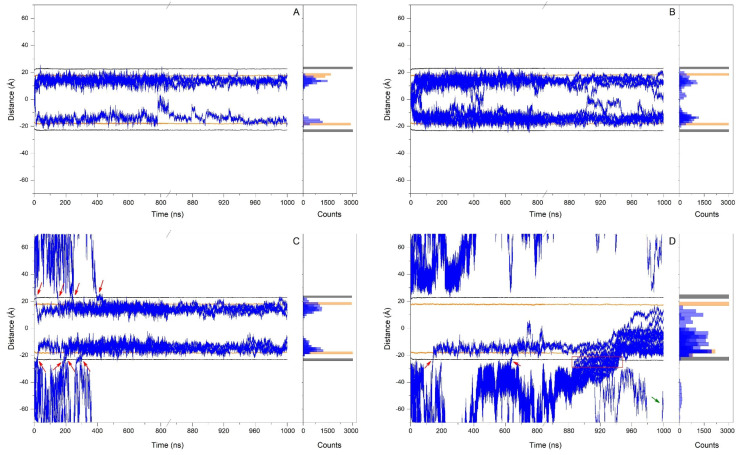
Time variation of the z-axis COM distance (middle of the membrane as a reference) for (**A**) system 1, (**B**) system 2, (**C**) system 3 and (**D**) system 4. Left panels represent the z-axis COM distance for the HNK molecules for the entire MD simulation, whereas the right panels represent the histograms corresponding to the z-axis COM distance for the last 30 ns of MD simulation time. The membrane upper and lower boundaries (z-axis distance of the phosphorous atoms of the phospholipids) is depicted in black, whereas the oxygen atoms of CHOL are depicted in orange. Red arrows in (**C**,**D**) mark the crossing of HNK molecules from the water layer into the membrane, green arrow in (**D**) marks the only HNK molecule which remained in the water solvent, whereas the red box in (**D**) marks the crossing of the HNK oligomer into the membrane.

As commented above, I have used in this work four distinct systems to determine the conduct of HNK with respect to the membrane. System 1 contained four molecules of HNK placed at the centre of the bilayer (Figure 2A). I have studied the behaviour of the z-axis centre-of-mass (z-COM) of the HNK molecules and compared it to the z-COMs of the phospholipid phosphorous and CHOL oxygen atoms in both layers (Figure 3A). All HNK molecules moved relatively fast, and at the conclusion of the simulation, i.e., 1 µs, all molecules had moved and reached a location a little lower than the oxygen atoms of CHOL but never above them (Figure 3A). Although one of the HNK molecules moved to the centre of the bilayer, it moved again to its previous position and remained there until the end of the MD (Figure 3A). For the last 30 ns of MD, the mean position of the phospholipid phosphorous atoms was 22.68 Å ± 0.1 Å, the mean position of the oxygen atoms of CHOL was 17.76 Å ± 0.2 Å, and the mean position of the z-COM of the HNK molecules was 14.22 Å ± 2.16 Å (Figure 3A, right panel). Remarkably, no HNK molecules clustered over time in system 1; all of them lasted in the monomeric state (Figure 2B). Consequently, the mean location of the molecules of HNK in this membrane system was in the upper part of the hydrocarbon chains, near the oxygen atoms of CHOL, below the phospholipid phosphorous atoms but near the phospholipid carbonyl groups.

System 2 had nine HNK molecules positioned at the middle of the bilayer (Figure 2B). The variation of their z-COM is shown in Figure 3B. Similarly to system 1, all HNK molecules moved relatively fast, and at the end of the simulation time, i.e., 1 µs, all molecules but one had moved and reached a location a little lower than the oxygen atoms of CHOL but never above them (Figure 3B). Several times, some HNK molecules moved to the centre of the bilayer but stayed there for not long and moved again to a position little below the oxygen atoms of CHOL (Figure 3B). It is then feasible for the HNK molecules to move along the palisade structure of the membrane formed by the hydrocarbon chains of the phospholipids, but their mean position should be below the oxygen atoms of CHOL. For the last 30 ns of MD, the mean position of the phospholipid phosphorous atoms was 23.1 Å ± 0.2 Å, the mean position of the oxygen atoms of CHOL was 18.1 Å ± 0.2 Å, and the mean position of the centre-of-mass of the HNK molecules was 13.1 Å ± 3.4 Å (Figure 3B, right panel). Interestingly, and similarly to the HNK molecules in system 1, no HNK molecules aggregated over time in system 2: all of them remained in the monomeric state (Figure 2B). Similarly to system 1, the mean position of the molecules of HNK in system 2 was in the upper part of the hydrocarbon chains, near the oxygen atoms of CHOL, below the phospholipid phosphorous atoms but near the phospholipid carbonyl groups. 

System 3 had eight HNK molecules positioned at the middle of the water layers, four in the upper water layer and four in the lower (Figure 2C). The variation of their z-COM is shown in Figure 3C. Similarly to systems 1 and 2, all HNK molecules moved relatively fast, and at the end of the simulation time, i.e., 1 µs, all molecules had reached a location a little lower than the oxygen atoms of CHOL but never above them (Figure 3C). As shown in the Figure, all the HNK molecules entered into the membrane at different times (red arrows, Figure 3C). Two of them crossed the membrane interphase very early in the simulation (at 14 ns and 23 ns). However, five of them crossed a little later, at about 147, 178, 188, 245 and 300 ns (Figure 3C). The last one to cross the interphase crossed at about 400 ns. In the end, all of them crossed and entered into the membrane, settling below the oxygen atoms of CHOL. This data tells me that the HNK molecules moved rapidly in water, and they entered into the membrane and localized in a defined place. Therefore, and again, the mean position of the molecules of HNK in this membrane system was in the upper part of the hydrocarbon chains, near the oxygen atoms of CHOL, below the phospholipid phosphorous atoms but near the phospholipid carbonyl groups. For the last 30 ns of MD, the mean position of the phospholipid phosphorous atoms was 23.0 Å ± 0.2 Å, the mean position of the oxygen atoms of CHOL was 17.9 Å ± 0.2 Å, and the mean position of the z-COM of the HNK molecules was 14.9 Å ± 2.6 Å (Figure 3C, right panel). Interestingly, and similarly to the HNK molecules in systems 1 and 2, no HNK molecules clustered over time; all of them lasted in the monomeric state (Figure 2C). 

System 4 had 18 HNK molecules positioned at the middle of the water layers, nine at the upper water layer and nine at the lower (Figure 2D). The variation of z-COM is shown in Figure 3D. Similarly to the previous systems, all HNK molecules moved relatively fast. However, along the MD simulation, different HNK molecules behaved distinctly. Two of the HNK molecules were independent from the bulk and crossed the membrane interphase at about 140 ns and 640 ns (red arrows, Figure 3D). The remaining ones formed a big agglomerate of HNK molecules, and all of them but one crossed the membrane interphase over a long time period, from about 880 ns to about 950 ns (red box, Figure 3D). One HNK molecule continued to be in the water solvent until the end of the MD simulation (green arrow, Figure 3D). The big HNK agglomerate was not completely static, since several molecules detached from it and crossed from one monolayer to the other (Figure 3D). In the end, one HNK molecule remained in the water layer, four HNK molecules were in the monomeric state and localized below the oxygen atoms of CHOL, and the remaining 13 were forming a big cluster of molecules approximately located at the centre of the phospholipid hydrocarbon layer (Figure 2D and Figure 3D).

I have measured the amount of hydrogen bonds for all systems and for the last 30 ns of MD. Practically no hydrogen bonds were found between the HNK molecules and either the lipids or the other HNK molecules. These data reflect the very low probability of these molecules of forming hydrogen bonds. However, as I have just shown for system 4, HNK, depending on concentration, formed high-order aggregates; these aggregates were not static, and the molecules that formed them could enter and leave them with relative ease. I have measured the number of contacts between the HNK molecules in system 4, and the results are shown in Supplementary Figure S3. As observed in the figure, some HNK molecules did not present any contact with the other HNK molecules (i.e., HNK molecule no. 4, being the one which remained in the water solvent, and HNK molecule no. 6, which remained in the monomeric state until the end of the MD). Molecules no. 5, 7 and 9 had very few of contacts because they were integrated into the aggregate in very little time. However, molecules no. 2, 8 and 15 had a large number of contacts because they were always united in the aggregate. The remaining HNK molecules had a lower number of contacts because they remained integrated in the aggregate for some time but not all the time. Clearly HNK in water can form clusters, likely in a concentration-dependent way; however, the HNK molecules in the aggregates are not static, and significantly, the formation of aggregates did not prevent HNK insertion into the interior of the membrane.

**Figure 4:**
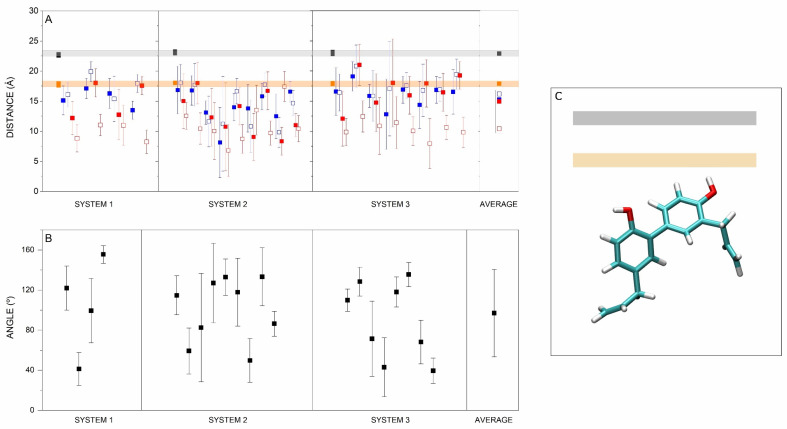
(**A**) Average z-COM for the oxygen (■□) and allyl carbon (■□) atoms of the HNK molecules of systems 1, 2 and 3, and (**B**) average angle between the two phenyl groups of the HNK molecules with respect to the membrane surface for systems 1, 2 and 3 (■), as indicated. System 1 comprised 4 molecules, system 2 had 9 molecules, and system 3 had 8 molecules, as indicated. The average data has been obtained for each one of the molecules and for the last 30 ns of MD simulation. (**C**) Representation of the global mean position of HNK in the membrane (grey and orange boxes delimitate the phospholipid phosphorous and oxygen atoms of CHOL, respectively). See text for details.

The HNK molecule is relatively rigid and does not cover the membrane width, and its extent in its most stretched conformation is approximately 12 Å. As I have commented above, the most favoured location of HNK is about 9 Å below the phospholipid phosphorous atoms, which define the membrane surface. Since HNK has two phenyl groups, it could be thought that it could have two completely distinct orientations in the membrane, either parallel or perpendicular to the membrane surface. In order to define in a better way the location and position of HNK in the membrane, I have measured the z-COM of the oxygen and allyl carbon atoms of the HNK in the monomeric state (systems 1, 2 and 3), and the results are shown in Figure 3A. Although there is a large scattering in the data (there were 21 HNK molecules in total), both oxygen atoms of HNK (~16.1 Å and ~15.3 Å) as well as one allyl carbon atom (~14.9 Å) were near the frontier demarcated by the oxygen atoms of CHOL (~17.8 Å), whereas the other allyl carbon atom (~10.3 Å) was below it (Figure 4A). I have also measured the angle formed by the two phenyl groups of HNK, and the results are shown in Figure 4B. Similarly to what was found previously, and taking into account that HNK is a relatively small molecule and that the data was collected for the last 30 ns of MD simulation, there was a large scattering in the data. The average global angle of HNK was found to be ~97° (Figure 4B). Taking into account the data of the z-COM of the individual atoms and the global average angle, the average global location and disposition of the HNK molecules should be similar to the one depicted in Figure 4C.

**Figure 5:**
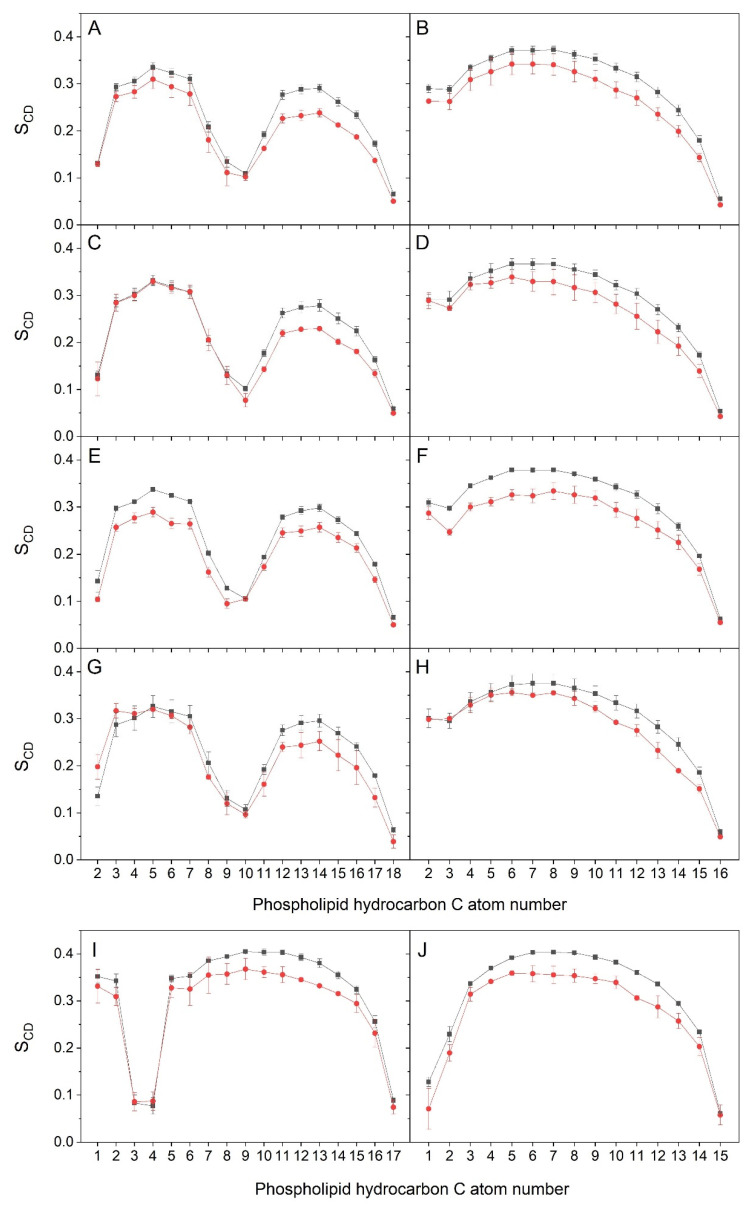
Average deuterium order parameter *S_CD_* calculated for the hydrocarbon chains of the phospholipids in systems 1, 2 and 3. The (**A**,**C**,**E**,**G**) oleoyl and (**B**,**D**,**F**,**H**) palmitoyl acyl chains of (**A**,**B**) POPC, (**C**,**D**) POPE, (**E**,**F**) POPS and (**G**,**H**) PI-3P, as well as the sphingosyl (**I**) and palmitoyl (**J**) acyl chains of PSM. The data correspond to the bulk phospholipid acyl chains (-■-) and the phospholipid acyl chains within 5 Å of HNK molecules (-●-). The analyses were carried out for the last 30 ns of simulation.

Bioactive molecules inserted into membranes can modify the phospholipid hydrocarbon chain order. Consequently, I investigated the effect of HNK on the hydrocarbon chain order by finding the deuterium order parameter, *S_CD_*, and averaging the data for systems 1, 2 and 3 as stated above (Figure 5). The *S_CD_* data of the acyl hydrocarbon chains of the bulk phospholipids, i.e., POPC, POPE, POPS, PI-3P and PSM, agreed with the profiles previously observed [60,61,62]. However, for those phospholipids which were near the HNK molecules, there were significant differences in the *S_CD_* profiles (remember that the data characterise the mean of 21 HNK molecules). The *S_CD_* values of the lipids near the HNK molecules were lower than the bulk ones (Figure 5). This diminution in the *S_CD_* values is more apparent at carbon atoms 6 to 14, where the HNK molecules are localised (see above). Therefore, monomeric HNK molecules insert well between phospholipid hydrocarbon chains and increase membrane fluidity, although they do not display a dramatic effect on the hydrocarbon chains’ anisotropy. It is important to note that the increase of membrane fluidity, that is, the increase of lipid disorder, exerted by HNK, in addition to having notable antioxidant properties, could modulate membrane structure and therefore affect the activity of certain proteins, so that the incorporation of HNK into membranes would not only affect the activity of these proteins but even cellular signalling.

The lipid mass densities for all four systems are shown in Supplementary Figure S4. All lipid profiles are basically symmetric between the two layers of the membrane, indicating comparable behaviour for all lipids. This would be the anticipated conduct, since I should remember that the lipid/HNK ratio of these systems is very high. A similar behaviour occurs with the phospholipid phosphorous atoms and the CHOL oxygen atoms, which are located at a similar distance in the four systems studied. The average mass densities of the HNK molecules encompass the CHOL molecules but are slightly below their oxygen atoms (Supplementary Figure S4). As commented above, all HNK molecules in systems 1, 2 and 3 were monomeric during all the MD simulations, but in system 4 many of the HNK molecules were forming a big agglomerate (see above). I have analysed the absolute mass density profiles of the HNK molecules in systems 1, 2 and 3 and compared them with the mass density profiles of the phospholipid phosphorous and CHOL oxygen atoms (Supplementary Figure S4I,J). The location of the HNK molecules was below the phospholipid phosphorous and slightly below the CHOL oxygen atoms. The average location of the HNK molecules was 14.6 Å, which can be compared with the average locations of the phospholipid phosphorous and CHOL oxygen, which were 23.0 Å and 18.1 Å, respectively (Supplementary Figure S4K).

The radial distribution function, g(r), i.e., the probability of the emplacement of each lipid in the membrane in relation to the HNK molecules, is shown in Supplementary Figure S5A. As stated above, I have calculated the average data from systems 1, 2 and 3, since they were the only ones whose HNK molecules were monomeric all the time. As observed in the figure, there is no clear distinction between the different lipid molecules: the most intense signals originated from POPE and POPC, which together with CHOL were the most abundant lipids in the system. The lowest intensity signal corresponded to PSM, whereas CHOL, PI-3P and POPS were a little more intense (Supplementary Figure S5A). I have also determined the nature and number of lipid molecules adjacent to the HNK molecules for the last 30 ns of the MD simulations for systems 1, 2 and 3, and the data are shown in Supplementary Figure S5B (observed data vs. the bulk quantity of the membrane lipids). There were no significant differences between the different lipids; if anything, a little more POPC than expected and a little less CHOL than expected were observed surrounding the HNK molecules. Overall, these data would imply that, in general, the HNK molecules do not have a significant preference for any one of the lipids in membranes. In this context, it is interesting to observe that HNK, in addition to not having any specific interaction with any of the membrane lipids, increases the fluidity of the membrane and forms aggregates as we have commented previously. These combined effects would imply that HNK might not be able to form pores in the membrane.

## 4. Conclusions

The results obtained in this work suggest that HNK, a pleiotropic bioactive hydrophobic molecule, tends to inserts spontaneously into biological membranes, locating near and below the CHOL oxygen atoms, amid the hydrocarbon chains of the phospholipids; while increasing membrane fluidity, HNK does not specifically interact with any membrane lipid, and, significantly, tends to associate in solution, establishing high-order aggregates, although such aggregation does not prevent it from inserting into the membrane. Some of the biological characteristics of HNK could be accredited to its aptitude to alter membrane biophysical properties, but the establishment of aggregate forms in solution might hamper its clinical use.

## Figures and Tables

**Table 1:** Systems and number of components used in this study. The NaCl concentration was 0.15 M. The production trajectories for each one of the systems were calculated for 1 µs. The total number of lipid molecules was 500, 250 per monolayer. The computing performance for each system was about 12 ns/day.

SYSTEMS	1	2	3	4
HNK	4	9	8	18
POPC	144	144	144	144
POPE	86	86	86	86
PI-3P	28	28	28	28
POPS	32	32	32	32
PSM	60	60	60	60
CHOL	150	150	150	150
No. LIPIDS	500	500	500	500
ATOMS	166,463	166,653	166,255	166,159
H_2_O	36,413	36,413	36,293	36,135
H_2_O/LIPID	72.5	72.8	72.6	72.3
Na^+^	219	219	219	218
Cl^−^	103	103	103	102
INITIAL DIMENSIONS x/y/z (Å)	128/129/120	128/129/120	128/129/120	128/128/120

## Data Availability

Data is contained within the article or Supplementary Material.

## Supplementary Materials

The following supporting information can be downloaded at: https://www.mdpi.com/article/10.3390/antiox13081025/s1. Figure S1: Time variation of membrane thickness for the whole simulation time (left panel) and corresponding histogram for the last 30 ns of MD simulation (right panel) for (A) system 1 (black), (B) system 2 (blue), (C) system 3 (red) and (D) system 4 (olive). The upper and lower data correspond to the phospholipid phosphorous and cholesterol oxygen atoms, respectively. Figure S2: Time variation of lipid areas for the whole simulation time (left panel) and corresponding histograms for the last 30 ns of MD simulation (right panel) for (A) system 1, (B) system 2, (C) system 3 and (D) system 4. Lipid areas correspond to POPC (black), POPE (blue), POPS (red), PI-3P (wine), PSM (olive) and CHOL (orange). Figure S3: Average number of molecular contacts between the HNK molecules for the last 30 ns of MD. Each number pertains to each one of the HNK molecules. Figure S4: Mass density profiles for the last 30 ns of the MD simulation for (A,B) system 1, (C,D) system 2, (E,F) system 3 and (G,H) system 4. The coloured lines correspond to the mass density profiles of the phosphorous atoms of POPC (black), POPE (blue), POPS (red), PI-3P (wine), PSM (olive), the oxygen atom of CHOL (orange), and the HNK molecules (magenta). (I) Average density, (J) normalized average density and (K) mean position for the phospholipid phosphorous atoms (black), the oxygen atom of CHOL (orange), and the HNK molecules (magenta) (systems 1, 2 and 3). Figure S5: (A) Average radial distribution function, g(r), for systems 1, 2 and 3. The lines correspond to POPC (black), POPE (blue), POPS (red), PI-3 P (wine), PSM (olive), and CHOL (orange). (B) Relationship between the total number of lipids in the membrane respective to the lipids surrounding the HNK molecules. The dotted line represents the equivalence between the observed vs. the real numbers. The analysis was carried out for the last 30 ns of simulation. Table S1: Membrane thickness (Å) and average molecular area (Å2) for the last 30 ns of the simulation for all the lipids in systems 1 to 4 studied in this work (rounded to the first decimal).

## Abbreviations

CHOL	Cholesterol
HNK	Honokiol (2-(4-hydroxy-3-prop-2-enylphenyl)-4-prop-2-enylphenol)
MD	Molecular Dynamics
NAMD	Nanoscale Molecular Dynamics
PI-3P	1-Palmitoyl-2-oleoyl-sn-glycero-3-phosphoinositol-3-phosphorous
POPC	1-Palmitoyl-2-oleoyl-sn-glycero-3-phosphocholine
POPE	1-Palmitoyl-2-oleoyl-sn-glycero-3-phosphoethanolamine
POPS	1-Palmitoyl-2-oleoyl-sn-glycero-3-phospho-L-serine
PSM	N-Palmitoyl-D-erythro-sphingosylphosphorylcholine
*S_CD_*	Deuterium order parameter
VDW	Van der Waals
VMD	Visual Molecular Dynamics
z-COM	z-axis Centre-of-Mass (z direction normal to the bilayer plane)
