# Supplementary material for: Localization and Aggregation of Honokiol in the Lipid Membrane
Source: Antioxidants (Basel). 2024 Aug 22;13(8):1025. doi: 10.3390/antiox13081025 (PMC11351574; doi:10.3390/antiox13081025)
Supplement: Supplementary file 1 [file antioxidants-13-01025-s001.zip › antioxidants-3096673-supplementary.pdf]

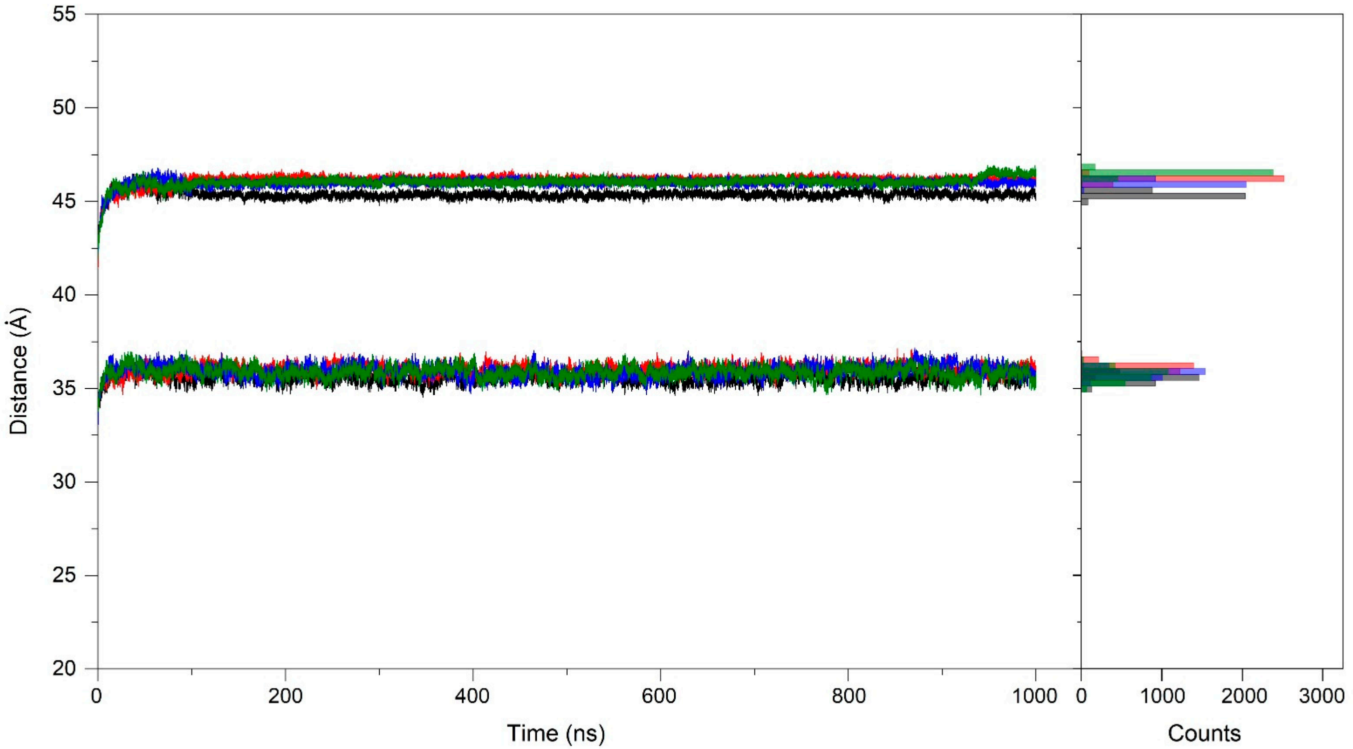

Supplementary Figure S1. Time variation of membrane thickness for the whole simulation time (left panel) and corresponding histogram for the last 30 ns of MD simulation (right panel) for (A) system 1 (black), (B) system 2 (blue), (C) system 3 (red) and (D) system 4 (olive). The upper and lower data correspond to the phospholipid phosphorous and cholesterol oxygen atoms, respectively.

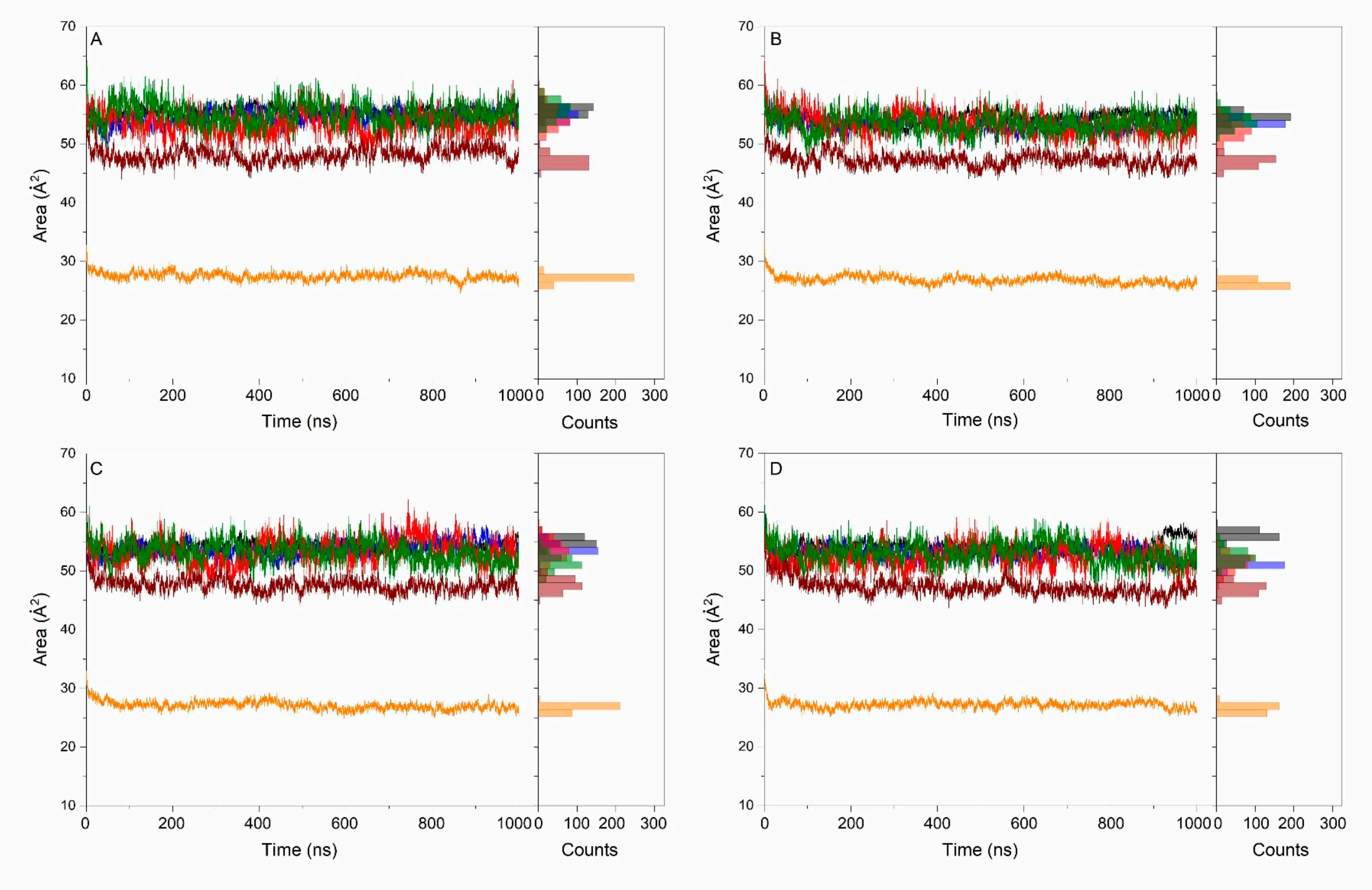

Supplementary Figure S2. Time variation of lipid areas for the whole simulation time (left panel) and corresponding histograms for the last 30 ns of MD simulation (right panel) for (A) system 1, (B) system 2, (C) system 3 and (D) system 4. Lipid areas correspond to POPC (black), POPE (blue), POPS (red), PI-3P (wine), PSM (olive) and CHOL (orange).

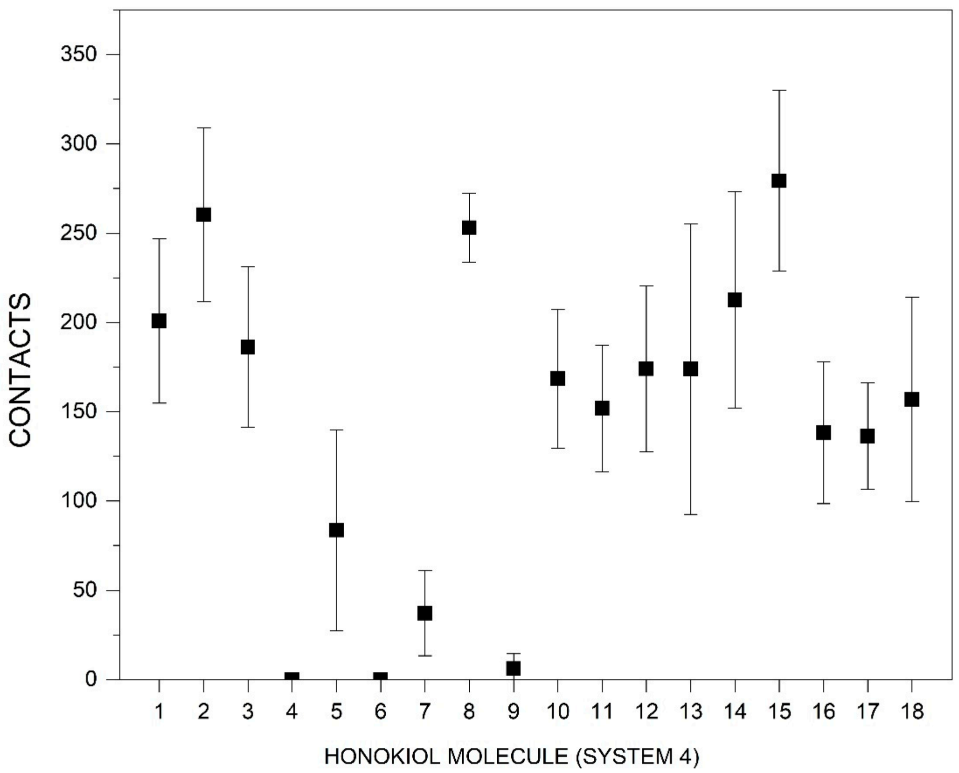

Supplementary Figure S3. Average number of molecular contacts between the HNK molecules for the last 30 ns of MD. Each number pertain to each one of the HNK molecules.

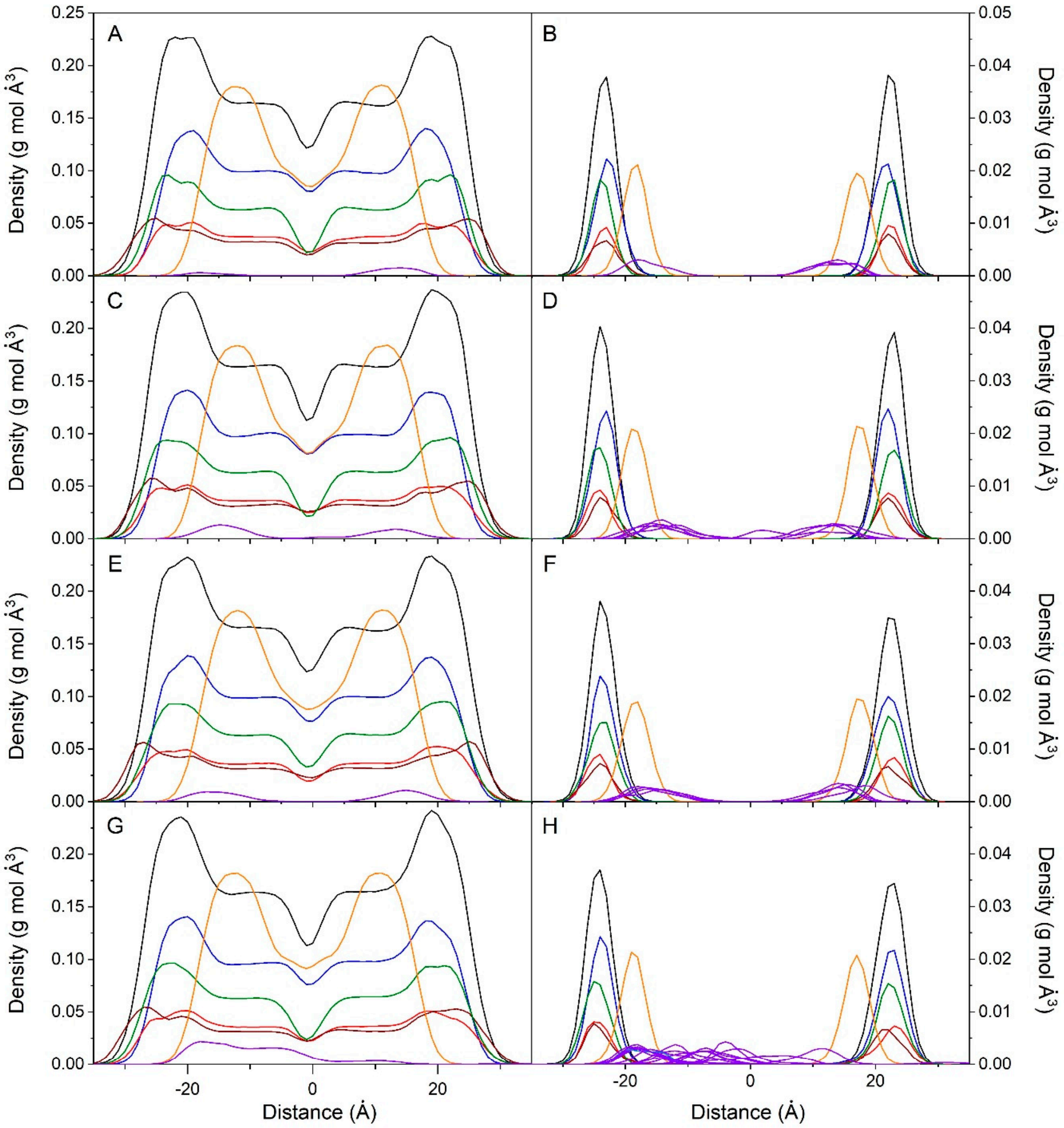

A
B
C
D
E
F
G
H
Density (g mol Å^3)
Distance (Å)
0.00
0.05
0.10
0.15
0.20
0.25
0.01
0.02
0.03
0.04
-20
0
20

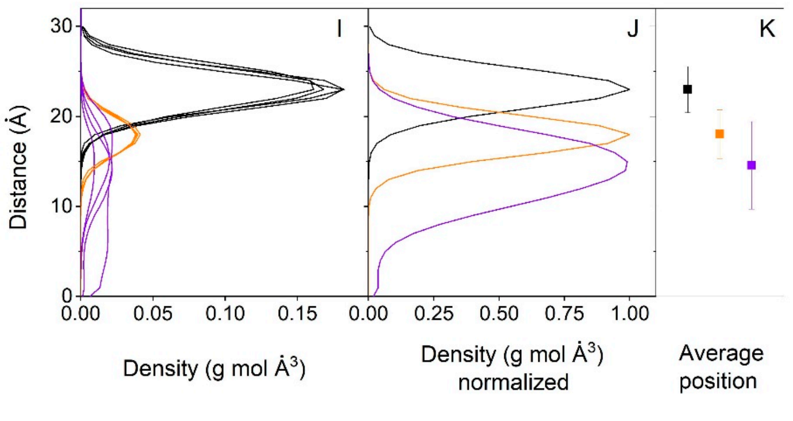

I
J
K
Distance (Å)
Density (g mol Å^3)
Density (g mol Å^3)
normalized
Average
position
0.00
0.05
0.10
0.15
0.25
0.50
0.75
1.00
0
10
20
30

Supplementary Figure S4. Mass density profiles for the last 30 ns of the MD simulation for (A, B) system 1, (C,D) system 2, (E,F) system 3 and (G,H) system 4. The coloured lines correspond to the mass density profiles of the phosphorous atoms of POPC (black), POPE (blue), POPS (red), PI-3P (wine), PSM (olive), the oxygen atom of CHOL (orange) and the HNK molecules (magenta). (I) Average density, (J) normalized average density and (K) mean position for the phospholipid phosphorous atoms (black), the oxygen atom of CHOL (orange) and the HNK molecules (magenta) (systems 1, 2 and 3). The mean position for each one.

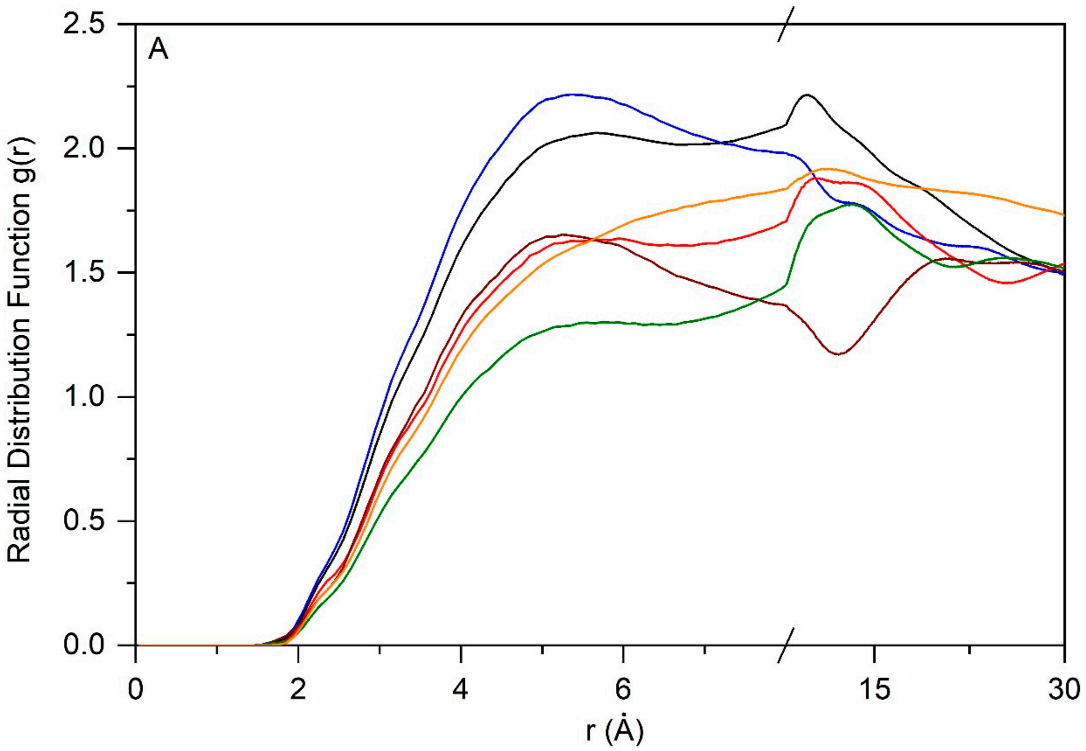

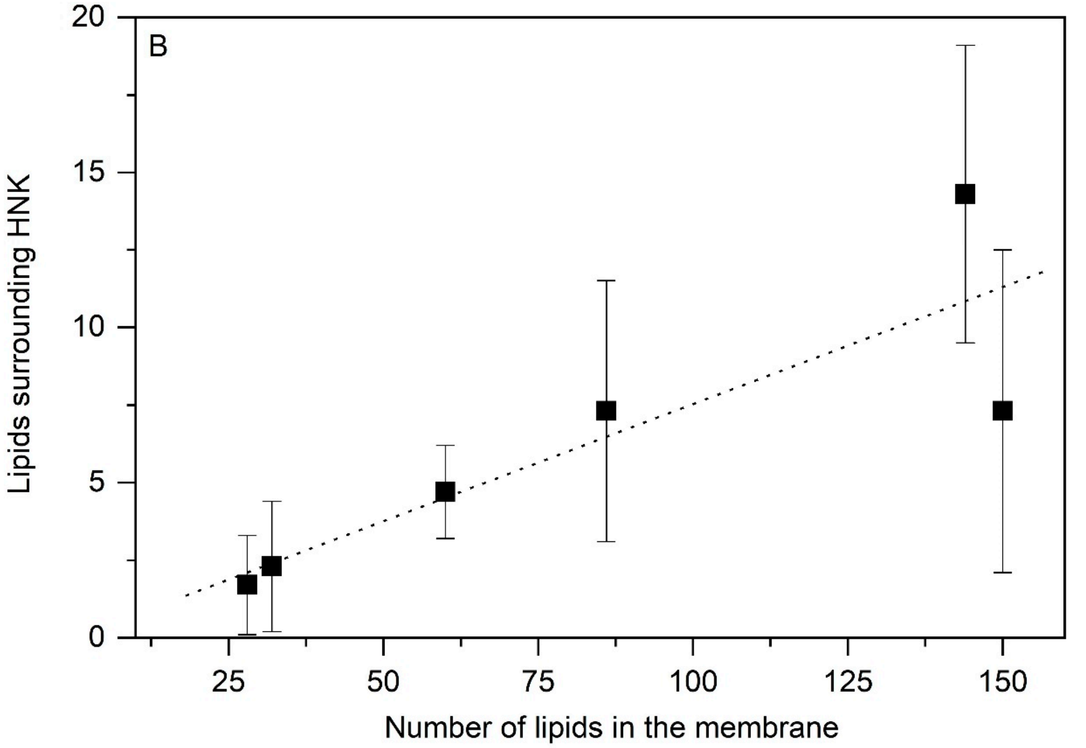

Supplementary Figure S5. (A) Average radial distribution function, g(r), for systems 1, 2 and 3. The lines correspond to POPC (black), POPE (blue), POPS (red), PI-3 P (wine), PSM (olive), and CHOL (orange). (B) Relationship between the total number of lipids in the membrane respect the lipids surrounding the HNK molecules. The dotted line represents the equivalence between the observed vs. the real numbers. The analysis was carried out for the last 30 ns of simulation.

Supplementary Table S1. Membrane thickness (Å) and average molecular area (Å^2) for the last 30 ns of the simulation for all the lipids in the systems 1 to 4 studied in this work (rounded to the first decimal).

SYSTEM	1	2	3	4
	MEMBRANE THICKNESS (Å)			
Phosphorous	45.8 ± 0.13	46.2 ± 0.1	46.0 ± 0.1	46.5 ± 0.1
Oxygen cholesterol	35.5 ± 0.23	36.1 ± 0.2	35.8 ± 0.2	35.8 ± 0.3
	MOLECULAR AREA (Å^2)			
POPC	55.7 ± 0.7	54.7 ± 0.6	55.0 ± 0.7	56.1 ± 0.7
POPE	55.0 ± 1.2	53.5 ± 0.8	53.5 ± 1.0	51.3 ± 0.7
POPS	54.6 ± 2.1	52.4 ± 1.4	53.5 ± 1.7	51.3 ± 1.7
PI-3P	55.7 ± 1.6	53.9 ± 1.3	51.4 ± 1.4	52.3 ± 1.3
PSM	47.0 ± 0.9	46.9 ± 0.7	47.7 ± 1.1	47.0 ± 0.9
CHOL	27.1 ± 0.5	26.3 ± 0.4	26.7 ± 0.5	26.5 ± 0.5
